# Using games to identify adolescents at risk for substance misuse—a proof-of-concept study

**DOI:** 10.3389/frhs.2026.1781703

**Published:** 2026-05-01

**Authors:** Kammarauche Aneni, Ching-Hua Chen, Jenny Meyer, Christina Mavromichali, Emmanuel Scaria, Gaoqianxue Liu, Youngsun T. Cho, Lynn Fiellin

**Affiliations:** 1 Yale Child Study Center, Yale School of Medicine, New Haven, CT, United States; 2 Biomedical Informatics and Data Science, Yale School of Medicine, New Haven, CT, United States; 3 IBM T.J. Watson Research Center, Yorktown Heights, NY, United States; 4 Biomedical Data Science, Dartmouth Geisel School of Medicine, Hanover, NH, United States

**Keywords:** adolescents, cognitive function, digital biomarkers, game-based assessment, in-game data, substance misuse, substance use screening

## Abstract

**Introduction:**

Early identification of adolescents at risk for substance use is critical for timely intervention. However, standard screening tools like CRAFFT (Car, Relax, Alone, Forget, Family and Friends, Trouble) and S2BI (Screening to Brief Intervention) face significant barriers, including adolescent disclosure reluctance, limited clinic privacy, and administrative challenges with paper-based forms. Digital games offer a promising alternative by generating behavioral data that may serve as digital biomarkers of substance use risk through engaging, low-burden gameplay. The objective of this proof-of-concept study was to explore the utility of game log data collected during gameplay to predict substance use.

**Methods:**

We analyzed game log data from 160 adolescents aged 11–14 years who played an HIV prevention game targeting high-risk behaviors including substance use, drawn from a larger randomized controlled trial (*N* = 333) conducted in schools. We extracted 240 gameplay-derived behavioral metrics reflecting executive function, decision-making, and inhibitory control—cognitive domains affected by substance use. Machine learning models (Support Vector Machine, Logistic Regression, Gradient Boosting, Neural Networks, Decision Tree, Random Forest) were trained to predict lifetime substance use and drug-refusal self-efficacy. A validation study with 36 newly recruited high school students tested associations with standardized measures of emotion regulation, impulsivity, and cognitive performance. Model performance was assessed using discrimination (e.g., AUC) and clinical utility (e.g., sensitivity) metrics.

**Results:**

Predictive accuracy across all models was insufficient for clinical translation. For drug use prediction, AUC (SD) ranged from 0.458 (0.11) to 0.593 (0.07) (mean AUC <0.6 across all models), sensitivity ranged from 0 to 0.265 (0.18), and F-1 scores ranged from 0.398 (0.0) to 0.482 (0.08). For drug-refusal self-efficacy prediction, AUC ranged from 0.425 (0.10) to 0.592 (0.09), sensitivity ranged from 0.386 (0.14) to 0.785 (0.19), and F-1 scores ranged from 0.431 (0.06) to 0.598 (0.07). No model achieved the minimum threshold (AUC ≥0.7) suggested for clinical utility.

**Discussion:**

Our study elucidates the challenges associated with extracting behavioral markers from naturalistic digital environments. We discuss the potential of game-based digital biomarkers as scalable, low-burden tools for screening and monitoring, and the limitations of our study that can inform future studies seeking to understand the feasibility of using in-game data as digital biomarkers of substance use risk in adolescents.

## 1 Introduction

Adolescent substance use remains a prevalent public health concern, with alcohol, cannabis, and nicotine among the most used substances in this age group (1). National surveys indicate that nearly 42% of U.S. high school seniors report having used alcohol in the past year and about 26% have used cannabis in that time (2). A significant minority of teens also experiment with other drugs; for example, 15% of high school students have ever used illicit drugs such as cocaine, heroin, or ecstasy, and a similar proportion have misused prescription opioids (3). This early initiation of substance use in adolescence is associated with numerous negative health and social consequences. Adolescents who engage in substance use often experience acute adverse outcomes including injuries, poorer academic performance, and mental health problems (3). Notably, substance use during adolescence can interfere with normal development; exposure to alcohol or drugs can alter critical brain maturation processes, leading to long-lasting changes in brain structure and function (4). Consequently, initiating substance use in adolescence heightens the risk for developing substance use disorders and other neuropsychiatric disorders in adulthood (4). In sum, adolescent substance use is common and carries both immediate health risks and enduring developmental impacts (4), underscoring the importance of early identification and intervention. This necessitates developing effective strategies for identifying youth at risk and monitoring their substance use behavior over time to mitigate harm.

Current approaches to identifying adolescent substance misuse rely largely on self-report screening questionnaires administered in clinical settings. While guidelines recommend routine screening of all adolescents for substance use, implementation in practice is inconsistent (5). Clinicians face practical barriers such as insufficient time during appointments and competing priorities, which limit their ability to thoroughly screen every adolescent patient (6). In one survey of primary care providers, “lack of time” was the most frequently cited barrier to adolescent substance use screening (6). Even when screening instruments are used, the clinic environment can hinder accurate disclosure. Adolescents may feel uncomfortable or unsafe reporting substance use on paper forms or face-to-face if they fear a breach of confidentiality or judgment from adults (7, 8). For instance, providers often note difficulties in finding private time to speak with teens one-on-one, especially when parents are present and unwilling to leave the exam room (6). This lack of privacy and fear of repercussions can lead adolescents to under-report or deny substance use. Moreover, many physicians report being unsure of how to manage a positive screen (e.g., what counseling or referrals to provide), which further reduces their motivation to screen routinely (6). These challenges mean that adolescents at risk for substance misuse may be missed by standard clinic-based, paper-and-pencil screening approaches (9). Screening inequities are also patterned by sociodemographic factors; a chart review of outpatient pediatric clinics found that providers were more likely to screen ethnic minority youth than white youth (10). This highlights that without standardized protocols, screening decisions may reflect implicit bias rather than universal need. There is a clear need for more engaging, scalable, objective, and effective methods to identify and track substance use risk and address present limitations.

Digital serious games have emerged as a promising tool to address some of these limitations in youth risk behavior assessment. Serious games are interactive games, including videogames, designed with a purpose beyond entertainment (such as education or health assessment) (11). They offer an engaging, familiar medium for adolescents; 95% of teens have access to a smartphone and about 85% play video games regularly (12, 13). Unlike one-off questionnaires, games can collect rich, objective data in real time as the user plays, creating opportunities for ongoing monitoring outside the clinic setting. A key advantage is that these data are captured passively and continuously during gameplay, potentially allowing for early warning signs of risk to be detected in real-world settings rather than relying solely on self-report which may contribute to under-reporting bias. Moreover, the interactive nature and perceived anonymity of gameplay may reduce stigma and enhance honest engagement among adolescents (14), which is a critical consideration for sensitive topics like substance use (14).

Within this framework, in-game behavioral data can serve as digital biomarkers—objective, quantifiable behavioral or physiological features that reflect health-related processes or risks. Because they arise from naturalistic behavior, these biomarkers can index neurocognitive and emotional functioning without relying on direct questioning (15, 16). Importantly, there are theoretical and empirical reasons to believe that game performance could reveal indicators of substance misuse risk. Specifically, playing video games recruits working memory for information retention, sustained and selective attention for monitoring game elements, strategic decision-making for evaluating options, and inhibitory control for regulating responses (17). Research has shown that impairment in cognitive functions increases vulnerability for substance misuse (18) and that youth who misuse substances tend to exhibit deficits in executive cognitive functions. For example, adolescent alcohol and marijuana users perform worse on tests of working memory, learning, and attention compared to non-users (18, 19). Such cognitive impairments can be especially pronounced with heavier use or earlier onset of substance use, and cognitive deficits may persist with chronic use (20). Furthermore, polysubstance use and more severe substance use disorders in adolescents have been linked to widespread executive dysfunction, including impaired inhibitory control, poorer decision-making, and reduced cognitive flexibility (4, 21, 22). Therefore, certain aspects of performance on cognitively demanding game tasks (for instance, making more impulsive errors on game tasks that require inhibitory control) may reflect underlying cognitive vulnerabilities associated with substance misuse (23). In essence, the game becomes an assessment tool, translating real-time behavioral data into insights about the player's cognitive profile. Early work suggests that game-based cognitive assessments can correlate with traditional neuropsychological tests, supporting their validity (23). By leveraging serious games and in-game digital biomarkers, it may be possible to create an engaging platform for real-time risk assessment and continuous monitoring of adolescents’ cognitive health and decision-making, thereby circumventing many of the barriers of clinic-based screening (9).

Although serious games and gamified assessments have been utilized in various youth health and cognitive domains, their application to substance misuse identification is still in its infancy. Prior studies on game-based interventions for adolescents have mostly focused on prevention or treatment outcomes (for example, improving knowledge, enhancing refusal skills, or supporting recovery) (24, 25). A recent systematic review of digital game interventions for adolescent substance use noted that games have been tested across prevention, treatment, and some assessment contexts (26). This review identified three games that focused on assessment, while the other 23 focused on treatment or prevention (26). Moreover, the assessment-focused game applications to date have generally involved measuring changes in attitudes or knowledge, rather than directly detecting substance use risk or impairment (26). To our knowledge, there are few if any published studies that specifically evaluate game-derived metrics as a tool for identifying youth with elevated substance use risk. In the broader field of cognitive assessment, game-based tools have shown promise for evaluating attention, memory, and related functions in children and adolescents (23), yet this approach remains underexplored for substance misuse screening. In short, a gap exists in the literature: while digital games have been harnessed for other behavioral health assessments and interventions, their potential role in objectively identifying adolescents prone to substance misuse has not been fully examined. This gap highlights the need for exploratory research to determine whether gameplay data can serve as an early indicator of substance use vulnerability.

Accordingly, we conducted a proof-of-concept study to test whether gameplay performance can be used to identify adolescents at elevated risk for substance use. Aim 1 was to train and evaluate machine learning models using in-game behavioral metrics that capture working memory, attention, decision making, and inhibitory control to predict binary outcomes of substance use and drug-refusal self-efficacy. Aim 2 was to externally validate the model from aim 1 using a new sample of adolescents, derive a digital biomarker score and examine whether these same gameplay metrics (using the biomarker score) are associated with performance on standardized cognitive tasks of working memory, inhibitory control, and selective attention. Together, these aims assessed whether machine learning applied to gameplay data can yield candidate digital biomarkers for early identification of substance-use risk in adolescents.

## 2 Materials and methods

The overall design has been previously described in our protocol paper (9). In aim 1, we used data from a prior randomized controlled trial (RCT) of an existing serious videogame, PlayForward, to develop and test a model to predict substance use among adolescents and to identify potential game-based digital biomarkers of adolescent substance use. In Aim 2, we recruited a new sample of high school adolescents to play the serious videogame with the goal to externally validate the model from Aim 1. The present study did not incorporate patient or public involvement into the design, conduct, or interpretation of this research.

### 1.1 Aim 1

#### 1.1.1 Data source and extraction

Data from a prior RCT testing a serious game, PlayForward, was used (27, 28). The RCT was conducted in schools in Connecticut, where 333 adolescents aged 11–14 years were randomized 1:1 to either PlayForward (*n* = 166) or attention matched control games (*n* = 167). Only participants randomized to PlayForward were included in the present study. Participants included adolescents who identified as Hispanic and non-Hispanic ethnicity; and Black, White and Other racial groups (27). The videogame collected in-game data related to play performance which were stored as log files (9, 29). Guided by the literature and the DSM-5 definitions of neurocognitive domains, 285 in-game metrics reflective of underlying cognitive function were extracted from the log files, stored in a CSV file and uploaded onto the Python Programming Language (version 3.8.2) (30) for analyses. These game metrics were identified from log data and inventory provided by the game developers which contained all possible metrics captured by the videogame. Members of the team played the videogame and identified potential metrics likely to employ cognitive processes (9). Examples of these in-game metrics included time spent completing specific game tasks which may be reflective of processing speed and working memory, correctly completing a task which may be reflective of working memory and decision making, and number of errors in a particular task, which may be reflective of impulsivity/inhibitory control (9) (see Table 1).

**Table 1:** Potential in-game biomarkers [reproduced from Aneni et al. (9)].

Minigame or subsection of minigame	Potential in-game biomarkers	Hypothesized neurocognitive domains (DSM-5^a^)
People sense minigame		
Deciding friends: Checking out each person’s information	- Time spent checking peer’s characteristics - Correctly sorting peers into the right social or friendship circles - Time spent completing social or friendship circles	- Working memory, attention, inhibitory control, and processing speed - Working memory and decision making - Working memory and processing speed
Accepting or rejecting invites	- Correctly accepting or declining invitations based on risk and social circles - Proportion of accepted unsafe invitations	- Working memory, decision making, and judgement - Decision making, and judgement
Refusal power minigame		
Think	- Correctly identifying type of peer pressure	- Decision making and inhibitory control
Prepare	- Correctly choosing reasons to say no	- Decision making and inhibitory control
Refuse	- Time spent completing sentence portion of minigame	- Working memory and processing speed
Priority sense minigame		
	- Number of times priority dropped to zero	- Working memory
Know sense minigame		
	- Total credibility score at the end of the minigame	- Working memory, decision making, and processing speed

^a^ DSM-5, Diagnostic and Statistical Manual, 5th edition.

#### 1.1.2 Outcomes

We had two outcomes: lifetime use of substances and self-efficacy to refuse drugs (9). Substance use was measured using the Youth Risk Behavior Survey (31) and assessed history of ever using alcohol, cigarettes, and other drugs (e.g., marijuana, tobacco). Past-30-day use of these substances were measured among those who responded yes to lifetime use. Substance use was coded as a binary variable with 1 for any use and 0 for no use (9). Self-efficacy was measured using the Drug Use Resistance Self-Efficacy scale (DURSE) (32) which assesses confidence in one's ability to refuse substances in a variety of risky situations. A sample question is, “How sure are you that you can refuse if an older friend, brother or sister offers you marijuana at a party and you do not want it?” Responses included four options which ranged from “not sure at all” to “definitely sure”. Higher scores reflected greater self-efficacy to refuse drugs. A binary self-efficacy score was derived based on the score distribution and adolescents were grouped in high vs. low self-efficacy based on their score (9).

#### 1.1.3 Statistical analysis

##### 1.1.3.1 Review of log files

We reviewed the log files of the 166 participants who played the videogame. After review, six files were excluded due to errors including incomplete game data. As such, log files from 160 participants were used for extraction of 285 in-game metrics (potential biomarkers) using R (33).

##### 1.1.3.2 Unsupervised feature selection

Unsupervised feature selection was applied to the 285 in-game metrics using a correlation matrix and variance threshold methods (34). Following data normalization, using the Min-Max method, we computed the correlation matrix and dropped in-game metrics that were highly correlated (correlation >0.95) with other metrics or variance less than 0.01, resulting in 240 in-game metrics for model development (see Supplementary Appendix 1).

##### 1.1.3.3 Model development

The final analytical dataset included 160 participants with 240 in-game metrics/features. We employed six machine learning (ML) binary classification models, including support vector machine (SVM), logistic regression, gradient boosting, neural network, decision tree and random forest. We discussed the justification for the selection of these models in our previously published protocol paper (9). Briefly, each of these modeling approaches has a different set of strengths and weaknesses, and the usefulness of an approach on a classification task is highly dependent on non-obvious attributes of a given data set.

We built a binary classification model for each of the two outcomes (drug use and self-efficacy). The hyper-parameters of the models were optimized via five-fold cross-validated grid-search over a parameter grid, with the AUC [Area Under the Receiver Operative Characteristic (ROC) curve] as the metric for model evaluation. We selected AUC as the scoring function for model optimization because it measures the performance of a model across multiple classification thresholds and is a more robust measure than accuracy when the data set is imbalanced. For both outcomes, model optimization was implemented using the GridSearchCV method provided by the scikit-learn Python library (35, 36). Code for data pre-processing and model development is available on GitHub (37). *Post-hoc*, we also explored if our models would be improved using class imbalance methods (e.g., SMOTE, undersampling).

##### 1.1.3.4 Model testing

By conducting 5-fold cross validation (38), we calculated statistics including AUC, sensitivity (true positive rate), specificity (true negative rate), precision, and F1 score (harmonic mean of precision and sensitivity) for each model. AUC values closer to 1 imply greater ability to discriminate between classes while AUC values closer to 0.5 imply a weaker ability to do so.

### 1.2 Aim 2

We recruited a new sample of adolescents with the goal of validating the model developed in Aim 1 and testing whether identified digital biomarkers correlate with cognitive function. We hypothesized that digital biomarkers will negatively correlate with cognitive function scores (9).

#### 1.2.1 Study procedures (recruitment, participants, source, recruitment and consent, data collection and storage)

Participants were high school students in Connecticut recruited between August 2023 and April 2024. High school students were selected because this aligns with the average age of onset of drug use (39, 40) and as such an important developmental period for identifying early risk of use. Eligibility for the study were as follows: “1) attend a high school in Connecticut, 2) be aged between 14 and 15 years, 3) be willing to sit for 60 min per session to play the game and 4) be able to provide assent and parent/guardian consent” (9). During school lunch periods, study staff distributed a brochure and flyers with a QR code to interested adolescents. Initially, adolescents who scanned the QR code were directed to complete a parent contact form for the study team to obtain parental permission via Qualtrics electronic signature. Following parental permission, adolescents were contacted for their assent. Adolescents who provided assent to participate in the study were invited to complete the baseline survey. Results of baseline survey were used to determine whether an adolescent was high vs. low risk (details below). Due to challenges with recruiting high-risk adolescents, we modified our eligibility criteria to increase the upper limit of the age range to 16 years and modified our procedures to screen adolescents for substance use prior to obtaining parental permission and adolescent assent. Initially, adolescents were deemed high and low risk by their baseline report of past month use of any substance (alcohol, marijuana, tobacco, prescription drugs, inhalants, illegal and synthetic drugs), plus >2 on the PHQ-2, and, >2 on the GAD-7 (9). However, due to poor recruitment of high-risk adolescents, we screened for substance use using an anonymous Qualtrics survey and defined high risk as having lifetime history of substance use (nicotine, tobacco, alcohol, cannabis, K2, spice, prescription pills or other illegal drugs). In the updated protocol, adolescents who completed the screener and reported any history of substance use were redirected to another Qualtrics survey to provide their contact information to obtain parental permission and assent. The Qualtrics screener was not linked to contact information survey to protect the privacy of the adolescent. Following screening and consent, adolescents completed baseline assessments and up to 2 h of gameplay per week until they completed the videogame. At baseline, all participants were given psychoeducational material from the National Institute on Mental Health on the teen brain and seeking help if needed.

PsyToolKit (41, 42) was used to complete baseline assessments and cognitive tasks. Data from PsyToolKit (used for baseline survey and cognitive tasks) and iPads (gameplay) were transferred to the Yale secure server at the end of each study day. Adolescents were compensated $50 for completing baseline assessments and $70 at the end of videogame completion to motivate completion of study procedures to ensure complete data collection among enrolled participants.

#### 1.2.2 Baseline assessments and cognitive tasks

Screening to Brief Intervention Scale (S2BI) (43): The S2BI is a 7-item substance use screening tool that assesses past year use of seven different groups of substances including alcohol, tobacco, marijuana, prescription drugs such as Adderall or pain medications not prescribed by a doctor, illegal substances such as cocaine, inhalants and synthetic drugs. Response options are on a 4-point scale ranging from “never” to “weekly”. For statistical analyses, data from the S2BI was used to define a binary drug use risk variable (no = never across all 7 items or yes = once or twice, monthly or weekly to any of the 7 items).

Drug Use Resistance Self-Efficacy Scale (DURSE): see description under “outcomes” in Aim 1 methods.

Difficulties in Emotion Regulation Scale—Short Form (DERS-SF) (44): The DERS-SF scale is an 18-item scale that assess challenges in regulating emotions. For example, “When I am upset, I become out of control”. Response options on a 5-point scale range from “almost never” to “almost always”. Some items were reverse coded such that higher scores reflected greater difficulty with regulating emotions. Mean scores are reported for the DERS-SF.

Urgency, Premeditation, Perseverance, Sensation Seeking, Positive Urgency, Impulsive Behavior Scale (Short UPPS-P) (45): The short form of the UPPS-P is a 20-item scale that assesses several aspects of impulsivity. A sample item includes, “When I am upset, I often act without thinking”. Response options range from “agree strongly” to “disagree strongly” on a 4-point Likert scale. Some items were reverse coded such that higher scores reflected greater problems with impulsivity. Mean scores of the UPPS-P were computed.

N-back task: The N-back task is a widely used cognitive assessment task to measure working memory (46). We employed the 2-back task. Participants were shown a series of letters and were instructed to press the *m* key on their keyboard if the letter was the same as the letter shown two trials back. PsyToolkit was used to administer this task (47). Participants were presented a total stimulus set of 15 letters where each stimulus was presented for 500 milliseconds. Participants had to respond in 3 s (47). Each participant had three blocks consisting of 25 trials (75 trials in total). We calculated the mean percentage error resulting from a false alarm (participant pressed the *m* key wrongly) and from a miss (participant failed to press the *m* key when presented with a letter matching the letter shown 2 trials back). Lower scores reflect better cognitive performance.

Stop-signal task: The stop-signal task assesses impulsivity (48). PsyToolkit was used to conduct this task (49). There were two blocks. In the first block, participants were shown a green left or right pointing arrow within a white circle and were instructed to press the *b* key when the left pointing arrow appears and the *n* key when the right pointing arrow appeared (49). Participants had to respond within 500 milliseconds. In the second block, participants were shown the arrows described above but with trials where the white circle turned red (49). Participants were instructed to press the corresponding key (*b* or *n*) when the circle was white (go trials) but do nothing (stop signal) when the circle turned red (no-go trials) (49). The white circle could be shown for up to 500 milliseconds while the red circle could appear between 100 milliseconds and 450 milliseconds following when the arrow is shown (49). We calculated the mean percentage error rate and mean response time in no-go trials. Lower scores reflect better cognitive performance.

Stroop task: The Stroop task assesses selective attention, processing speed and cognitive flexibility (50). PsyToolkit was used to conduct this task (41, 51). Participants were shown four colors names (blue, green, red, yellow) in different print colors (e.g., blue printed in yellow color). Participants were instructed to press the letter key that corresponded to the print color (e.g., if blue is printed in yellow color, the correct key to press would be “y”). Participants were shown 40 trials with varying number of compatible (color name matches print color) ranging from 8 to 12 and incompatible trials (color name does not match print color) ranging from 28 to 32 (51). We calculated the mean response time during compatible and incompatible trials and the Stroop effect (mean response time during incompatible trials minus the mean response time during compatible trials). Lower scores reflect better cognitive performance.

#### 1.2.3 Statistical analysis

##### 1.2.3.1 Statistical tests

Given that our model metrics (AUC) from the validation tests was well below 0.7 across all models (see results below), we did not proceed with our *a priori* plan to compute a digital biomarker score using in-game metrics to predict cognitive function (9). Instead, post-hoc, we explored the association between substance use risk and cognitive functioning using *t*-test differences of means and correlations (52, 53). Analyses were conducted using Stata 18 (54).

#### 1.2.4 Power

Although we did not compute digital biomarker scores for reasons cited above, our *a priori* sample size of 30 was based on an expected correlation of 0.5 between in-game performance scores and validated measures of cognitive function similar to prior validation studies comparing game-based cognitive assessment to standardized assessment (23). We calculated that 30 participants will provide 80% power at the 0.05 two-sided significance level to detect a moderate correlation of 0.5 between in-game performance markers and cognitive function.

## 2 Results

### 2.1 Aim 1

#### 2.1.1 Participant characteristics

Data from 160 participants from the prior videogame RCT was used for model development and internal validation. Table 2 shows the age and sex distribution as well as the proportion of adolescents endorsing drug use and high confidence in refusing drugs (self-efficacy). About 1/3rd of adolescents reported history of drug use while half reported high self-efficacy to refuse drugs.

**Table 2:** Characteristics of Aim 1 participants.

Characteristics	Frequency *N* = 160	Percent (%)
Age		
11	50	31.3
12	48	30.0
13	34	21.3
14	28	17.5
Sex		
Male	87	54.4
Female	73	45.6
Drug use		
Low risk	107	66.9
High risk	53	33.1
Self-efficacy to refuse drugs		
Low self-efficacy	78	48.8
High self-efficacy	82	51.3

#### 2.1.2 Model results

Table 3 shows the metrics for machine learning models for the drug use outcome. Overall, model performance was not impressive with AUC and F1 scores <0.6 across all models. This, combined with the observation that several of the models in Table 3 have very high specificity (close to 1) and very low sensitivity (close to zero), suggest that these models are trivially predicting the negative drug use class simply because the outcome is more prevalent in the data, rather than capturing meaningful relationships between the outcome and the in-game features.

**Table 3:** Drug use model metrics.

Model	AUC (Mean)	SD	Sensitivity (Mean)	SD	Specificity (Mean)	SD	F-1 Score	SD	Precision	SD
SVM	0.536	0.129	0	0	1	0	0.401	0.005	0.334	0.008
Logistic Reg	0.527	0.086	0.038	0.047	0.954	0.05	0.426	0.052	0.483	0.211
Gradient Boosting	0.593	0.073	0.055	0.109	0.963	0.046	0.439	0.098	0.437	0.213
Neural Networks	0.547	0.092	0	0	0.991	0.018	0.398	0.004	0.333	0.007
Decision Tree	0.458	0.109	0.265	0.176	0.729	0.088	0.482	0.077	0.483	0.08
Random Forest	0.533	0.116	0	0	0.991	0.018	0.398	0.004	0.333	0.007

SVM, support vector machines.

Table 4 shows the model metrics for the self-efficacy outcome. Similar to the drug use model, model performance was unimpressive with AUC and F1 < 0.6 across all models.

**Table 4:** Self-efficacy model metrics.

Model	AUC (Mean)	SD	Sensitivity (Mean)	SD	Specificity (Mean)	SD	F-1 Score	SD	Precision	SD
SVM	0.592	0.092	0.622	0.101	0.578	0.078	0.598	0.07	0.601	0.068
Logistic Reg	0.525	0.063	0.549	0.12	0.514	0.077	0.529	0.074	0.532	0.072
Gradient Boosting	0.503	0.082	0.785	0.189	0.256	0.089	0.481	0.078	0.584	0.176
Neural Networks	0.453	0.078	0.6	0.109	0.463	0.073	0.526	0.038	0.534	0.04
Decision Tree	0.425	0.101	0.386	0.144	0.497	0.158	0.431	0.062	0.439	0.064
Random Forest	0.463	0.082	0.538	0.081	0.464	0.132	0.496	0.08	0.5	0.079

SVM, support vector machines.

### 2.2 Aim 2

#### 2.2.1 Study participants

Ninety-five students expressed interest in the study and completed the online screening, of which 36 were enrolled and completed baseline assessments. Of the 36 enrolled in the study, 31 participants completed gameplay. The average number of minutes among participants who completed gameplay was 368 min (range = 180–570 min). Baseline data was used for *post-hoc* analysis. Table 5 shows the baseline characteristics of study participants. About 2/3rds of the participants were aged 15 years and over. One quarter of the participants identified as Hispanic. The most common drugs misused by adolescents included alcohol, marijuana and prescription drugs which included stimulants and opioids. About one third of participants reported past year use of drugs resulting in being classified as high-risk.

**Table 5:** Characteristics of Aim 2 participants.

Characteristics	Frequency *N* = 36	Percent (%)
Age		
14	11	30.6
15	22	61.1
16	3	8.3
Sex		
Male	18	50
Female	18	50
Race		
Asian or Pacific Islander	4	11.1
Biracial/ Multiracial	6	16.7
Black or African American, non-Hispanic	14	38.9
Middle Eastern/North African	2	5.6
White non-Hispanic	9	25
Ethnicity		
Hispanic	9	25
Non-Hispanic	27	75
Classes of drugs		
Alcohol	6	16.7
Marijuana	5	13.9
Tobacco	1	2.8
Prescription drugs (e.g., pain meds, Adderall)	5	13.9
Illegal drugs (e.g., cocaine, ecstasy)	1	2.8
Inhalants	0	0
Herbs or synthetic drugs (e.g., salvia, K2 or bath salts)	0	0
Drug use		
Low risk	26	72.2
High risk	10	27.8
Self-efficacy to refuse drugs		
Low self-efficacy	13	36.1
High self-efficacy	23	63.9

#### 2.2.2 Results

Table 6 shows associations between baseline characteristics and drug use. Adolescents reporting drug use were more likely to be older, report a lower efficacy to refuse drugs and greater impulsivity. No differences between adolescents in the low vs. high-risk group were found across race, ethnicity, working memory, inhibitory control, selective attention or processing speed.

**Table 6:** Differences in baseline characteristics and cognitive functions by drug use risk outcome.

	Substance use risk		
Characteristics or outcomes	Low risk *N* = 26 (%)	High risk *N* = 10 (%)	*T* or *χ*^2^ (*p*-value)
Demographic characteristics			
Mean age in years (SD)	14.7 (0.5)	15.1 (0.7)	**−2.13** (**0.041)**
Female sex (%)	50	50	0.00 (1.000)
Race (%)			4.09 (0.393)
Asian or Pacific Islander	11.5	10.0	
Biracial/ Multiracial	15.4	30.0	
Black or African American, non-Hispanic	46.2	20.0	
Middle Eastern/North African	7.7	0.0	
White non-Hispanic	19.2	40.0	
Ethnicity			0.18 (0.667)
Hispanic/Latinx	23.1	30.0	
Non-Hispanic/non-Latinx	76.9	70.0	
Other baseline outcomes (self-report scales)			
Mean self-efficacy (SD)	94.4 (5.5)	86.3 (9.9)	**3.11** (**0.004)**
Mean emotion regulation score (SD)	38.2 (10.4)	40.9 (8.1)	−0.73 (0.472)
Mean impulsivity score (SD)	40.7 (4.8)	45.8 (6.1)	**−2.66** (**0.012)**
Cognitive tasks scores (objective measures)			
Working memory (N-back task)			
- False alarms mean PE (SE)	15.3 (4.3)	4.7 (1.6)	1.50 (0.144)
- Missed trials mean PE (SE)	35.2 (4.1)	40.6 (7.1)	−0.67 (0.509)
- Compatible trials mean response time (SE)	721.8 (38.3)	815.6 (83.6)	−1.15 (0.257)
Inhibitory control (Stop-signal task)			
- No-go trials mean PE (SE)	29.5 (3.3)	27.9 (2.5)	0.29 (0.773)
- No-go trials mean response time (SE)	367.6 (7.6)	386.2 (5.5)	−1.45 (0.157)
Selective attention/processing speed (Stroop task)			
- Compatible trials median time (SE)	915.64 (30.5)	943.3 (67.6)	−0.43 (0.669)
- Incompatible trials median time (SE)	992.0 (22.6)	952.2 (50.0)	0.84 (0.409)
- Stroop effect (SE)	76.3 (19.1)	8.9 (30.8)	1.47 (0.150)

pe, percentage error, SD, standard deviation; SE, standard error.

Bolded: *p* < 0.05.

Table 7 shows associations between baseline characteristics and self-efficacy to refuse drugs. Adolescents with low self-efficacy to refuse drugs were more likely to report drug use, greater impulsivity and poorer emotion regulation. There were no differences in cognitive function outcomes by self-efficacy.

**Table 7:** Differences in baseline characteristics and cognitive functions by self-efficacy outcome.

	Self-efficacy to refuse drugs		
Characteristics or outcomes	High self-efficacy *N* = 23	Low self-efficacy *N* = 13	*T* or *χ*^2^ (*p*-value)
Demographic characteristics			
Mean age in years (SD)	14.7 (0.5)	15 (0.7)	1.75 (0.09)
Female sex (%)	10 (43.5)	8 (61.5)	1.08 (0.30)
Race (%)			3.30 (0.51)
Asian or Pacific Islander	3 (13.0)	1 (7.7)	
Biracial/ Multiracial	4 (17.4)	3 (23.1)	
Black or African American, non-Hispanic	10 (43.5)	4 (30.8)	
Middle Eastern/North African	2 (8.7)	0 (0)	
White non-Hispanic	4 (17.4)	5 (38.5)	
Ethnicity			0.36 (0.55)
Hispanic/Latinx	5 (21.7)	4 (30.8)	
Non-Hispanic/non-Latinx	18 (78.3)	9 (69.2)	
Other baseline outcomes (self-report scales)			
Mean drug use score (SD)	7.0 (0.2)	8.5 (1.5)	**4.66** (**0.0000)**
Mean impulsivity score (SD)	39.6 (4.3)	46.5 (4.9)	**4.42** (**0.0001)**
Mean emotion regulation score (SD)	36.4 (9.2)	43.5 (9.6)	**2.17** (**0.04)**
Cognitive tasks scores (objective measures)			
Working memory (N-back task)			
- False alarms mean PE (SE)	15.2 (4.8)	7.4 (2.5)	−1.16 (0.25)
- Missed trials mean PE (SE)	35.3 (4.3)	39.1 (6.3)	0.51 (0.61)
- Compatible trials mean response time (SE)	718.3 (42.8)	798.8 (63.8)	1.07 (0.29)
Inhibitory control (Stop-signal task)			
- No-go trials mean PE (SE)	28.1 (3.7)	30.7 (1.9)	0.50 (0.62)
- No-go trials mean response time (SE)	369.8 (8.6)	378.1 (5.5)	0.68 (0.50)
Selective attention/processing speed (Stroop task)			
- Compatible trials mean PE (SE)	964 (30.8)	980.6 (49.2)	0.29 (0.77)
- Incompatible trials mean PE (SE)	1,034.9 (24.6)	1,020.8 (42.0)	−0.31 (0.76)
- Stroop effect (SE)	70.4 (21.0)	40.3 (28.8)	−0.86 (0.40)

pe, percentage error; SD, standard deviation; SE, standard error.

Bolded: *p* < 0.05

As described in our protocol (9), we planned to compute digital biomarkers scores using the logistic regression model, but the resulting metrics from Aim 1 indicated a poor fitting model, precluding the utility of applying model to Aim 2 data.

## 3 Discussion

### 3.1 Principal findings

We conducted a study to explore the utility of using in-game metrics for predicting drug use risk among adolescents. Using six machine learning classification models, our results revealed sub-optimal metrics with low AUC (0.43-0.59), F1 and precision scores across all models. Furthermore, the drug use models had high specificity (often >0.90) at the cost of sensitivity. In other words, the models tended to label nearly all participants as low-risk (negative class), potentially due to class imbalance in the training data, although strategies to address class imbalance (over- and under-sampling techniques) (55) did not improve these results. For example, the logistic regression classifier identified only ∼4% of true past-year substance users (sensitivity ∼0.04) while correctly classifying ∼95% of non-users (specificity ∼0.95). Similarly, models predicting low self-efficacy to refuse drugs performed no better than chance (all AUC <0.6), with inconsistent sensitivity-specificity tradeoffs across algorithms, suggesting that the utility of using the non-specific game metrics to screen for substance use may be limited. Below we discuss potential reasons for our negative findings and future directions.

First, the sample of adolescents across both studies (Aims 1 and 2) were relatively low risk (past-year use of substances). Since recruitment was school-based in contrast to a treatment setting, this may have contributed to recruiting fewer participants with extensive or heavy drug use histories. Adolescents with only experimental or infrequent use might not yet exhibit the neurocognitive deficits seen in more chronic users (19). Notably, cognitive impairments related to substance use tend to be most pronounced in youths with heavier or earlier-onset use (56, 57). These deficits are especially evident in teens with diagnosable substance use disorders or polysubstance use, who show widespread impairments in inhibitory control, decision-making, and cognitive flexibility (18, 22, 56, 57). In our sample, the absence of strong group differences in cognitive performance could reflect that most “high-risk” participants had relatively mild use that had not substantially affected executive functions. This low baseline risk may have diminished the signal that the models could detect. Using a game-based assessment in a more severe or clinical sample may yield larger cognitive differences, but that would undermine the tool's value as an early screener. Balancing this trade-off remains a challenge. To determine if indeed game-based performance captures drug-related risks, future studies with a higher risk sample would be needed.

Second, the outcome definition in this study encompassed multiple substances with diverse cognitive effects. The “high-risk” group included any past-year use of various drugs (from cannabis to prescription stimulants), which may have opposing impacts on cognition. For example, stimulant medications (even if misused without prescription) can acutely improve attention and working memory in the short term (58), whereas cannabis use is associated with worsening of working memory and inhibitory control (59). Thus, aggregating different substances into one outcome may have obscured performance patterns or even produced counterintuitive results at the group level. In fact, we observed a surprising finding that adolescents who reported drug use showed a better Stroop task performance (a smaller Stroop interference effect) compared to drug-free peers although this difference did not reach statistical significance. Specifically, the high-risk group exhibited minimal slowing on incongruent Stroop trials compared to congruent trials, whereas low-risk participants showed the expected reduction in speed under interference. One plausible explanation is that some high-risk youths were misusing stimulants (obtained from peers or sources other than prescribed by a doctor), which may have enhanced their selective attention during testing, in contrast to cannabis users who might have been underrepresented or whose impairments were averaged out. This substance heterogeneity likely introduced noise and bias in the data, masking any consistent cognitive signature of “at-risk” adolescents. A more granular, substance-specific approach might be necessary to detect subtle cognitive differences relevant to that particular drug. Again, the utility of a game-based tool that assesses only one specific substance may have limited value as a screener since the goal is to broadly identify risk across substances to deliver early intervention. However, such a tool, if able to accurately and specifically identify risk for a particular substance, may have much more utility for monitoring during treatment.

### 3.2 Limitations

This study has several limitations that temper the interpretation of our findings. First, the cognitive performance metrics extracted from gameplay were non-specific and potentially influenced by many factors other than substance use. We selected in-game behaviors (e.g., errors, response times, task completion rates) hypothesized to engage working memory, attention, and impulse control. However, these same cognitive processes are impaired in a variety of non-substance-related conditions such as attention-deficit/hyperactivity disorder (ADHD) (60) and depression (61). We did not screen out or control for such comorbid conditions, which may have been present in our sample across both aims. It is possible that some participants classified as “low risk” for substance use had underlying ADHD or mood disorders that adversely affected their game performance (e.g., slower responses or more impulsive errors), whereas some “high-risk” substance-using teens did not. This may have introduced noise and even reverse expected group differences, thereby confounding our results. Future studies might mitigate this by assessing common comorbidities as part of screening to ensure subgroups have similar distributions of these comorbid conditions. Ultimately, the lack of cognitive specificity in our game metrics makes it difficult to attribute poor performance uniquely to substance use risk, since other conditions could produce similar gameplay patterns.

Second, the serious videogame used in this study was not originally designed as a game-based cognitive testing battery. While we hypothesized that certain in-game behaviors aligned with executive function, the game's primary purpose was educational/behavioral change, not assessment. Participants could employ varied strategies to succeed in the game that may not isolate a particular cognitive ability in the way that a standardized neuropsychological test would. In contrast, dedicated game-based assessment tools explicitly embed cognitive tasks into the gameplay and have demonstrated much stronger validity. For instance, serious games that incorporate classic cognitive tests have shown good concordance with traditional lab measures of those functions (23). One benefit of using games that are not specifically designed to measure cognitive function is the potential scalability of using games that adolescents already enjoy playing and the ecological validity they provide since they would closely mirror real life experiences. In a prior systematic review we conducted evaluating the validity of games for assessing cognitive function, of the 18 games included in our review, only one of these games (Minecraft) was not specifically developed to assess cognitive function (23). In our review, the study that assessed the validity of Minecraft to measure cognitive function, specifically assessed spatial ability and abstract reasoning (fluid intelligence) and found significant correlations between specific Minecraft tasks and these cognitive functions when measured using standardized measures (23). It is possible that performance on games not specifically designed to assess cognitive functions may reflect more broad cognitive functions such as abstract reasoning in contrast to narrow functions such as working memory (62). By using an existing educational game, our approach may have sacrificed some precision regarding narrow cognitive functions—the metrics were essentially *post hoc* proxies rather than purpose-built measures. This limitation may have reduced the sensitivity of the metrics we examined. To better understand the utility of existing games for assessing cognitive functions, future studies are needed to further investigate if these games (those specifically developed to assess cognitive functions) are better at assessing broad cognitive functions. Furthermore, developers should consider building games with cognitive assessment in mind or inserting well-validated cognitive task modules into the game environment, to ensure that the data collected are tightly linked to the constructs of interest (in this case, neurocognitive markers of substance risk).

Moreover, our sample was drawn from a single region and was moderate in size, further limiting representativeness. These factors highlight a fundamental tension: a screening tool should ideally detect early, subtle risk in the general population, yet studying it in an easily accessible school sample entails a low base rate of the very condition (significant substance impairment) that it seeks to detect. Future research should attempt to include more diverse populations and possibly oversample youths with known higher risk profiles to better train and test models, while still aiming for an application that works in routine, community settings. Additionally, given the sample size, extensive subgroup analysis across various demographic groups was not done. Despite these limitations, it is important to recognize that the concept of game-based risk assessment remains novel, and the limitations identified can inform improvements in both study design and game development for this purpose.

### 3.3 Future directions

On the basis of the above, future work should aim to clarify substance-specific cognitive signatures rather than treating adolescent use as a homogenous category using larger samples of adolescents. For example, a cannabis-only cohort would allow targeted predictions about deficits in working memory and inhibitory control that are repeatedly observed in adolescent and young-adult users, including recent neuroimaging evidence of altered working-memory activation patterns (63). By contrast, a nicotine-only cohort could test for short-term attentional or working-memory enhancement and longer-term neurodevelopmental vulnerability unique to cholinergic modulation during adolescence (64). In both cases, excluding polysubstance users at the design stage would reduce cognitive signal cancellation and improve assay sensitivity, though with the caveat that early experimentation often spans multiple substances and transitions over time (65–67), limiting the utility of a single-substance screener.

Future studies may also benefit from a design that leverages cue-reactivity (68). Individuals with substance use disorders show robust craving and attentional bias when exposed to drug-related cues in immersive environments; virtual-reality studies report increased craving to smoking and other drug cues and emerging evidence that cue-exposure responses predict relapse risk (69). In this vein, gamified tasks could embed brief drug-cue probes to identify shifts in in-game behavior (70). Research on attentional bias in gaming has shown that people with problematic gaming or addiction tend to respond differently to game-related cues. For example, showing slower reaction times or automatic approach tendencies when those cues appear (71). These same behavioral paradigms, such as the addiction Stroop task, could be adapted to include substance-related cues within serious games, enabling the assessment of how strongly players’ attention or actions are influenced by drug-relevant stimuli (72).

### 3.4 Conclusions

This proof-of-concept pilot study provides one of the first examinations of the utility of game-based performance data to cognitive measures of substance use risk among adolescents. Using games to assess cognitive risk for substance use can mitigate several barriers to assessing substance use risk among adolescents, but only if these accurately reflect this underlying risk. Overall, our results suggest limited utility, although these results must be interpreted cautiously considering several factors that may explain our negative results. We have discussed several ways in which future studies can build on this study to better inform the utility of games for assessing substance use risk among adolescents.

## Data Availability

The raw data supporting the conclusions of this article will be made available by the authors, without undue reservation.
